# Posttraumatic Stress Disorder and Nonadherence to Treatment in People Living With HIV: A Systematic Review and Meta-analysis

**DOI:** 10.3389/fpsyt.2020.00834

**Published:** 2020-08-19

**Authors:** Jianhua Hou, Jiangning Fu, Siyan Meng, Taiyi Jiang, Caiping Guo, Hao Wu, Bin Su, Tong Zhang

**Affiliations:** ^1^Center for Infectious Diseases, Beijing Youan Hospital, Capital Medical University, Beijing, China; ^2^Beijing Key Laboratory for HIV/AIDS Research, Beijing, China; ^3^Institute of Psychology, Chinese Academy of Sciences, Beijing, China; ^4^School of Public Health, Yale University, New Haven, CT, United States

**Keywords:** posttraumatic stress disorder, people living with HIV, meta-analysis, antiretroviral therapy adherence, systematic review

## Abstract

**Background:**

Posttraumatic stress disorder (PTSD) is a commonly reported and serious complication among people living with HIV (PLWH). PTSD may significantly increase unintentional non-adherence to antiretroviral therapy. In this systematic review and meta-analysis, we aimed to pool the observational studies exploring the association between PTSD and medication adherence among PLWH.

**Methods:**

Comprehensive searches were conducted in PubMed/Medline, Web of Science, PsycINFO, Google Scholar, and ProQuest to identify relevant articles and dissertations. A random effects meta-analysis with inverse variance weighting was used to summarize the odds ratio (OR) across studies. Meta-regression and subgroup analyses were also carried out to assess the moderation effects for potential factors.

**Results:**

By synthesizing 12 studies comprising 2489 participants, the pooled odd ratio of non-adherence to antiretroviral therapy was 1.19 (95% confidential interval (CI), 1.03–1.37, *p* = 0.02). No significant publication bias was detected by Egger’s test (Intercept = 0.842, *p* = 0.284). Factors moderating the association were mean age of participants, depression adjustment, and depression (all *p* < 0.05).

**Conclusions:**

This meta-analysis supports that PTSD is related to adherence in PLWH. The hypothesized mechanisms (avoidant behavior and cognitive impairment) underlying this association need further investigation. Overall, this study highlights that clinicians should thoughtfully integrate timely mental health intervention into routine care.

## Introduction

Compared with the general population, posttraumatic stress disorder (PTSD) is a commonly reported psychiatric disease among people living with HIV (PLWH) (1–4). The estimated prevalence of PTSD in PLWH was 28% worldwide, and PTSD is a frequent encountered psychiatric comorbidity in HIV primary care setting (5). PLWH with PTSD symptoms may be tremendously affected by unaffordable public health cost, disabling psychological distress, worsened quality of life, and even increased odds of suicide (6, 7). Moreover, an increasing amount of evidence suggests that PTSD like other commonly reported mental health disorders, may accelerate HIV disease progression characterized by significant CD4 decline (8, 9). Although the mechanism underlying PTSD and HIV disease progress remains far from understood, non-adherence to antiretroviral therapy (ART) may represent one such mechanism (9).

One previously published systematic review has summarized the impact of different mental health disorders including PTSD on ART non-adherence (10). No quantitative analysis was adopted in that review. As far as we know, no previous meta-analytic study has assessed the relationship between PTSD and ART adherence for PLWH. Moreover the mixed results of original articles also necessitate the quantitative analyses (9, 11–21). Such a quantitative method also enables us to clarify whether the association is moderated by different population- and study- based factors.

Previous studies exploring the association between PTSD and ART nonadherence adjusted several demographic and psychological confounders. A large number of studies reported that depression and substance were associated with lower level of ART adherence in PLWH (22–27). We thus hypothesized that depression and substance use might be added risk factors for PLWH with PTSD. Age, a commonly reported demographic confounder, is associated with cognitive impairment, especially the worsened prospective memory which is related to higher missing doses of medication among the participants with chronic disease (28–31). Gender disparity may also exist. Brown et al. (19) found significant gender disparity in the association between trauma experience and ART adherence (19). Thus we tried to figure out the moderator effects of the commonly reported confounders in previous original studies and interpret the potential mechanism.

In summary, we conducted a systematic review and meta-analysis to quantitatively pool the observational studies exploring the association between PTSD and ART adherence among PLWH. We hypothesized PLWH with PTSD would be more likely to be non-adherent to their ART. We also tried to figure out potential moderators that influence the association.

## Methods

This meta-analysis complied with the Meta-analysis Of Observational Studies in Epidemiology (MOOSE) checklist (32).

### Search Strategy

Comprehensive searches were conducted in PubMed/Medline, Web of Science, PsycINFO, Google Scholar, and ProQuest to identify relevant articles and dissertations. The search terms included an intersection of PTSD- and participant- terms. PTSD terms included “Posttraumatic stress disorder,” “PTSD,” and “trauma.” Participant terms included “HIV,” “AIDS,” and “human immunodeficiency virus.” All searches were restricted to English articles and dissertations. Additional searches were conducted in the reference lists of included articles. The detailed information for searching process is attached in **Supplementary Table 1**.

### Selection Criteria

Studies were considered as eligible if they met all of the following inclusion criteria:

P: people living with HIV;
I: not available;
C: PTSD vs no PTSD;
O: the association of PTSD and ART adherence (coefficient or odd ratio);
S: cohort or cross-sectional study

### Study Selection and Data Coding

We used Endnote version 7 to assist the process of study selection. First, titles and abstracts were preliminarily screened to exclude irrelevant studies by JHH and TYJ. Second, full-text versions were assessed independently to ensure that all inclusion criteria were met by JHH and TYJ. Disagreement in the process of study selection was resolved by discussion with TZ. The two researchers used standardized Microsoft Excel spreadsheets to extract the following information: authors, year of publication, country of study, number of participants, study design, age, gender, education level, PTSD assessment, and adherence assessment.

### Data Analyses

The primary outcome of interest was the association between PTSD and ART adherence among PLWH. Risk estimates were standardized to express odds ratios of non-adherence. A random effects meta-analysis with inverse variance weighting was used to summarize the odds ratio (OR) in CMA 2 (Comprehensive Meta-Analysis version 2, Biostat, Englewood, NJ). The extent and significance of between-study heterogeneity was assessed by I-square and Cochrane Q test, respectively (33). Egger’s linear regression test were used to assess the potential publication bias (34). For categorical moderators, sub-group analyses were adopted to compare different stratifications. The categorical moderators included type of trauma (HIV-related vs. mixed), adherence measurement (objective vs. subjective), study quality (low vs. high), years since HIV diagnosis (fewer than or equal to five years vs. more than five years) and depression adjustment (Yes vs. No). For continuous moderators, meta-regression analyses were used to explore linear association. The continuous moderators included age, female proportion, proportion of depression, and substance use. All analyses with *p*<0.05 were considered as statistical significance.

### Quality Assessment

We used the Agency for Healthcare Research and Quality (AHRQ) checklist with a maximum score of 11 to assess the study quality for observational studies (35). Each item is worth one point. The original categorization for this checklist were 0–4 (high-risk), 5–8(moderate), and 9–11(low-risk). We used stricter criteria for study quality categorization in this meta-analysis. Studies were considered as low-risk if they scored more than 8 while other studies with a score lower than or equal to 8 were considered as high-risk. We also calculated the pooled results stratified by study quality to check the robustness of our results.

## Results

### Characteristic of Included Studies

**Figure 1:**
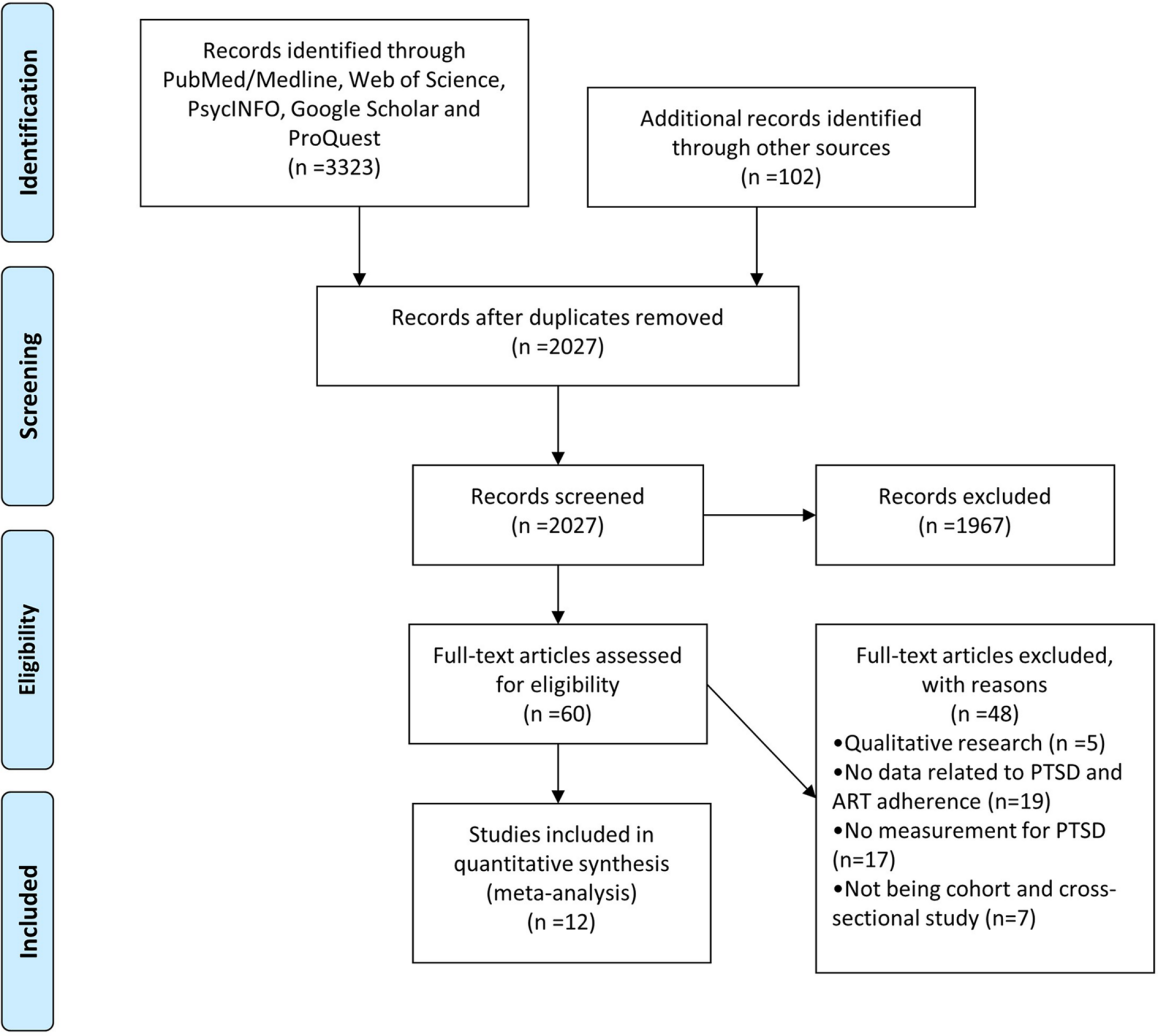
The flowchart of included studies.

The comprehensive search yielded 3425 items (**Figure 1**). Twelve studies comprising 2489 participants met the inclusive criteria (**Table 1**). The mean age ranged from 38 to 55 (n = 10) and the female proportion ranged from 0 to 70.5% (n = 9). Three studies (3/12, 25%) were categorized as high-quality. The detailed information for quality assessment is shown in **Supplementary Table 2**.

**Table 1:** Characteristic of included articles.

Study name	N	Age	Gender (female)	Time since diagnosis	Type of trauma	PTSD prevalence	PTSD measurement	Nonadherence rate	Adherence measurement	Study quality
Boarts et al. (11)	57	NR	18%	NR	HIV diagnosis, interpersonal trauma, disaster, accident, imprisonment/torture and other distress events	42.1%	PDS	NR	Self-report from AACTG	7
Brown et al. (19)	402	45	34.9%	10	Accidental/disaster-related trauma	70.2%	LEC	NR	Self-report(one 5-Likert question)	10
Delahanty et al. (12)	110	40.77	17.3%	8.06	HIV diagnosis	NR	IES	NR	Self-report from AACTG	6
Ebrahimzadeh et al. (9)	220	38	41.4%	5.9	HIV-related trauma	19.1%	Mississippi (Echelle) PTSD measure	64.5%	Self-report from AACTG	7
Halkitis et al. (13)	180	55.4	0	18.4	HIV-related trauma	NR	TATC	NR	Self-report from AACTG	9
Keuroghlian et al. (14)	38	45.8	NR	NR	HIV diagnosis	44.7%	IES	42.1%	self-report (1 question from AACTG)	6
Negi et al. (20)	305	>40 (42%) < = 40 (58%)	41.6%	NR	Earthquake and HIV-related trauma	43.9%	PCL-S	7.9%	Self-report from AACTG	7
Nilsson Schönnesson et al. (15)	193	43	25%	0.5–17	HIV diagnosis	28%	IES	37%	Self-report from AACTG	7
Sauceda (21)	146	42.2	NR	9.52	HIV diagnosis, childhood sexual assault, and other life events	NR	IES-R	NR	Visual analog scale, open-ended questions (frequency of missed meds, missed appts)	6
Vranceanu et al. (16)	156	42.1	23.7%	NR	HIV diagnosis	21%	SPAN	NR	Electronic pill bottle cap	6
Wagner et al. (17)	214	44	NR	NR	HIV diagnosis, disaster, accident, sexual assault and other distress events	38%	PDS	NR	Electronic pill bottle cap	7
Whetten et al. (18)	468	41.8	70.5%	4.4	HIV diagnosis and lifetime events	NR	PCL	17.3%	Three-item self report	8

### Assessments of PTSD and ART Adherence

All studies adopted self-report scales. The self-report scales and questionnaires included Impact of Event Scale (IES) or its revised version (IES-R); the PTSD Checklist (PCL) or its Stressor Specific (PCL-S); the Trauma Awareness and Treatment Center (TATC) PTSD Scale; the Posttraumatic Diagnostic Scale (PDS); the Startle, Physiological arousal to reminders, Anger, and Numbness (SPAN) and Mississippi (Echelle) PTSD measure.

Ten studies (10/12, 83.3%) used self-report, and four articles (2/12, 16.7%) used various objective adherence measurement. Seven studies adopted Medication Adherence Questionnaire of AIDS Clinical Test Group (ACTG). Two studies adopted a three-item self-report questionnaire or a five-point Likert scale, respectively. Sauceda’s team systematically assessed the missing doses in past 7 days and past month, due medication doses, frequency of taking all scheduled doses for one month, ability to take all medicine in five questions. Two studies used the pill-counting method by the following formula: Medication adherence=number of pills actually used by the patient/number of pills that the patient should have used*100%.

### Overall Association Between PTSD and ART Adherence

**Figure 2:**
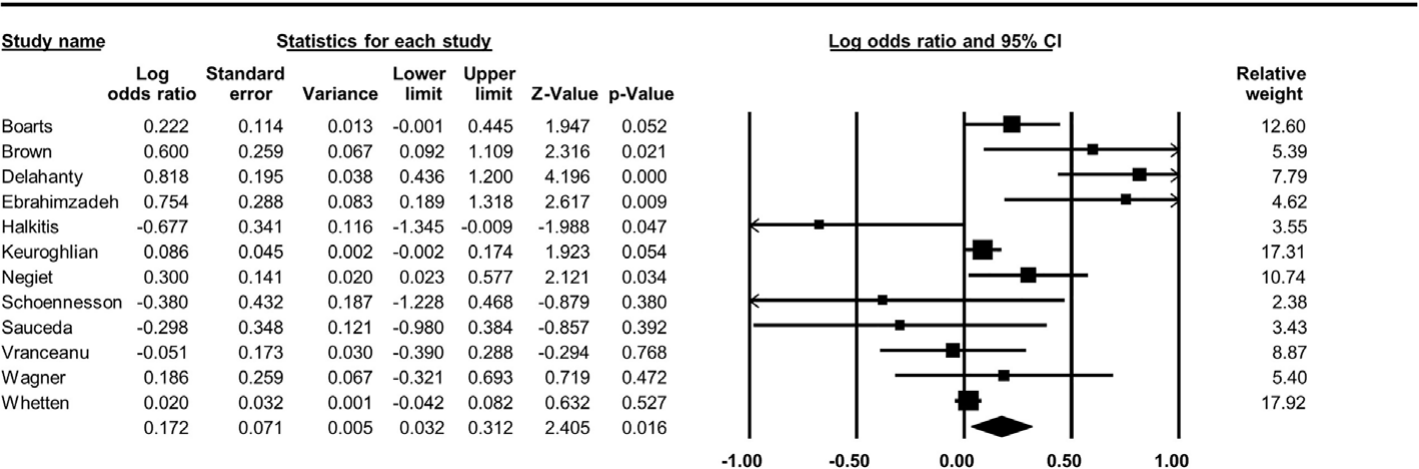
The forest plot for the association between PTSD and ART adherence for PLWH.

**Figure 3:**
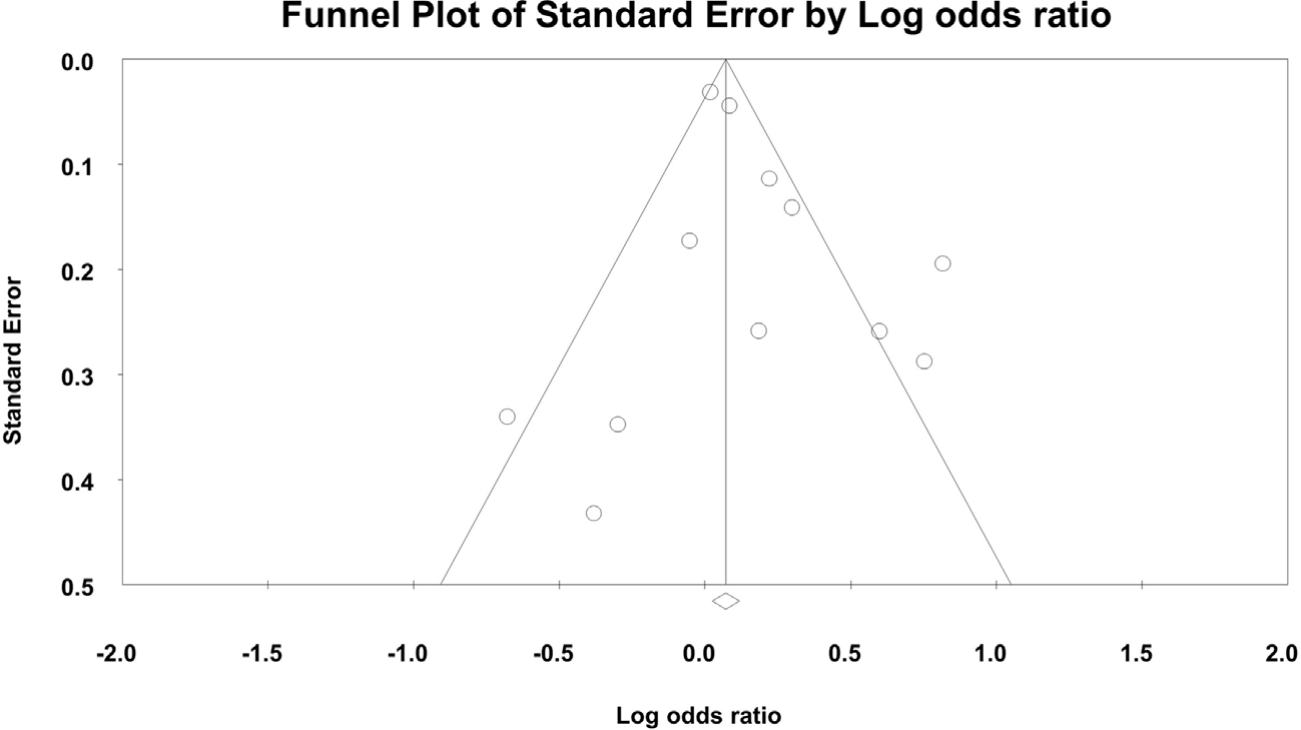
The funnel plot for the association between PTSD and ART adherence for PLWH.

By synthesizing 12 studies, the pooled odds ratio was 1.19 (95% CI, 1.03–1.37, *p* = 0.02, **Figure 2**), indicating increased odds of non-adherence to ART for participants with PTSD. The between-study heterogeneity was significant and large (Q(11) = 39.29, I-square = 72.01, *p*<0.01). No significant asymmetry was detected by Egger’s test (Intercept = 0.842, *p* = 0.284, **Figure 3**).

### Factors Related to the PTSD-Adherence Association

Mean age of participants was significantly related to the PTSD-adherence association (Q(1) = 5.59, *p* = 0.018), indicating that compared to younger participants, older PLWH was associated with lower odds of non-adherence for individuals with PTSD. Adjustment of depression status was also significantly related to PTSD-adherence association (Q(1) = 5.48, p = 0.019), indicating that compared to depression-unadjusted studies, PLWH was associated with lower odds of non-adherence for individuals with PTSD in depression-adjusted studies. The level of depression was associated with higher odds of non-adherence among participants with PTSD (Q(1) = 7.594, p = 0.006). Other factors did not significantly moderate this association. Only three studies reported substance use proportion, and we thus did not carry out moderator analysis for this variable (15–17). The detailed information for moderator analyses is shown in **Table 2**.

**Table 2:** Subgroup and meta-regression analyses of moderators.

Factors	Number of Studies	Stratification	OR	95% CI	Q	*p*
Type of trauma	12				0.007	0.932
	6	HIV-related	0.167	−0.102 to 0.436		
	6	Mixed	0.183	−0.065 to 0.431		
Adherence	12				0.562	0.453
	10	Subjective	0.195	0.04–0.35		
	2	Objective	0.039	−0.337 to 0.416		
Country development	12				0.381	0.537
	9	Developed	0.137	−0.081 to 0.356		
	3	Less developed	0.263	−0.069 to 0.595		
Study quality	12				0.764	0.382
	9	Low	0.222	0.17–0.426		
	3	High	0.041	−0.309 to 0.391		
Depression adjustment	12				5.48	0.019
	9	Yes	1.07	1.008–1.137		
	3	No	1.474	1.178–1.844		
Time since diagnosis	7				0.373	0.541
	3	<5	0.161	−0.362 to 0.684		
	4	> = 5	0.378	−0079 to 0.834		
Age	10		−0.065	−0.119 to −0.11	5.59	0.018
Female proportion	9		0.003	−0.01 to 0.016	0.155	0.693
Depression proportion	6		1.155	0.334–1.976	7.594	0.006

## Discussion

As far as we know, this is the first systematic review and meta-analysis that explores the PTSD-ART adherence association among PLWH. The present meta-analysis of 12 studies found that there was elevated odds of ART non-adherence amongst HIV-infected participants with PTSD. Although the effect size for this association was small, from the perspective of public health, the result is still meaningful in making public health policy in terms of high prevalence (28%) of PTSD among PLWH worldwide (5). Moreover, there was a significant effect toward this association among younger participants and depression-unadjusted studies.

Underlying the relationship between PTSD and ART adherence in PLWH relates to cognitive impairments and PTSD symptoms. PLWH may already have potential impairment in multi-cognitive domains that have been linked to ART non-adherence (36). Specifically, PLWH is profoundly associated with prospective memory deficit which is a strong risk of concurrent problems ranging from medication non-adherence to employment (31). PTSD-caused cognitive decline may also lead to regular forgetting of medication in many chronic diseases (such as cardio-vascular diseases) (37, 38) and may further lead to treatment failure. Future prospective cohort studies are thus needed to verify the interaction of HIV and PTSD on non-adherence among PLWH and to explore the mediation effect of cognitive impairment in PTSD-adherence association.

Additionally, avoidance, a specific PTSD symptom, may lead to intentional non-adherence (39). HIV-related medication may remind participants may bring back memories of the traumatic life event, and re-experience illness belief and death fear (40, 41). In the other hand, PTSD may also lead to low trauma coping self-efficacy and self-control (42). Thus, PLWH with PTSD may skip the medication by avoidance of traumatic memories and a sense of futility.

As aforementioned, our moderator analyses also shed lights on roles of participants’ age and depression. Within this narrow age range in this meta-analysis (38–55.4), older participants may develop more systematic coping strategies to confront the traumatic events. Conversely, biological ageing process and HIV infection are associated with severe prospective memory deficit which may lead to medication nonadherence (31, 43). We are not sure whether the association pattern between PTSD and adherence is consistent throughout the whole life span. Future longitudinal studies should verify the association in different age groups. Depression significantly moderated the association, indicating depression may act as an added risk factor associated with non-adherence. A recent meta-analysis comprising nine studies demonstrated that PLWH with depression were 14% less likely (pooled odds ratio [OR], 0.86; 95% CI, 0.71–1.05) to use ART than those without depression (44). In the meanwhile, though there are not enough studies exploring other psycho-social factors in PTSD-adherence literatures, these factors (anxiety, stigma, and substance use) may also be related to ART adherence (27, 45–47). As a result of the complexity of the influential path, the association will require further clarification by structural equational modeling (SEM).

Several limitations should be mentioned. First, limited articles in potential moderators (for instance, anxiety and substance use) make it hard to draw conclusion on all related factors. In addition, the reported association in this study should also interpreted in cautious due to limited studies. Second, the interactive effects between all these factors necessitate more complicated mathematical modeling methods (such as SEM). Third, only ¼ articles were categorized as high quality, which may result in potential bias. Furthermore, there was a high between-study heterogeneity. However, no group difference was detected between high vs. low quality studies. Next, we cannot draw causal relationship from this meta-analysis and exclude potential confounding effects due to the cross-sectional nature of included studies. Future studies adopting randomized controlled trials are also needed to verify the causal relationship between PTSD and ART nonadherence. Furthermore, only studies in English were accepted. And the cutoff for the p-value was not corrected for multiple comparisons, which increases the risk of false positive.

## Conclusion

In conclusion, this is the very first meta-analysis suggesting that PTSD is related to ART nonadherence among PLWH. The age of participants and depression adjustment our statistical models moderated this association. Some hypothesized mechanisms (avoidant behavior and cognitive impairment) underlying this association need further investigation. PTSD is one of the most commonly reported mental disorders among PLWH, so clinicians should thoughtfully integrate timely mental health intervention into routine care.

## Data Availability Statement

The data supporting this meta-analysis are from previously reported studies and datasets, which have been cited. The processed data are available from the corresponding author Dr. Tong Zhang (zt_doc@ccmu.edu.cn) upon request.

## Author Contributions

JH conceptualized the study. JH and TJ searched the literature, selected studies, and extracted the data. JH and JF contributed to the analysis and interpretation of the data and provided important scientific input. JH analyzed the findings and wrote the first draft of the manuscript with input from BS. SM, and CG revised the first draft with concrete contribution. HW and TZ supervised the study. All authors contributed to the article and approved the submitted version.

## Funding

This work was supported by the National Science and Technology Major Project: the National 13^th^ Five-Year Grand Program on Key Infectious Disease Control (2017ZX10202102-005-003 to BS, 2018ZX10301-407-005 and 2018ZX10302103-001-003 to TJ, 2018ZX10715-005-002-002 to CG, and 2017ZX10202101-004-001 to TZ); the Beijing Municipal of Science and Technology Major Project (D161100000416003 to HW, and D161100000416005 to CG); and the Beijing Key Laboratory for HIV/AIDS Research (BZ0089). The funders had no role in study design, data collection and analysis, decision to publish, or preparation of the manuscript.

## Conflict of Interest

The authors declare that the research was conducted in the absence of any commercial or financial relationships that could be construed as a potential conflict of interest.
