# Supplementary material for: Posttraumatic Stress Disorder and Nonadherence to Treatment in People Living With HIV: A Systematic Review and Meta-analysis
Source: Front Psychiatry. 2020 Aug 19;11:834. doi: 10.3389/fpsyt.2020.00834 (PMC7466667; doi:10.3389/fpsyt.2020.00834)
Supplement: Supplementary file 1 [file Table_1.docx]

**Table 1 Full searching strategies**

| Sources | Search date | Key words |
| --- | --- | --- |
| Medline/PubMed | Initial search:12.3.2019  Update: 2.20.2020 | We randomly combined two key words from two sets of searching terms, respectively. The two sets of searching terms included participant- term (HIV OR AIDS) AND PTSD-terms (PTSD OR post-traumatic stress disorder OR trauma) in title/abstract. |
| Web of science | Initial search:12.5.2019  Update: 2.16.2020 | We randomly combined two key words from two sets of searching terms, respectively. The two sets of searching terms included participant- term (HIV OR AIDS) AND PTSD-terms (PTSD OR post-traumatic stress disorder OR trauma) in topic. |
| PsycINFO | Initial search:12.10.2019  Update: 2.17.2020 | PsyINFO is a commonly used database to search psychology articles. We randomly combined two key words from two sets of searching terms, respectively. The two sets of searching terms included participant- term (HIV OR AIDS) AND PTSD-terms (PTSD OR post-traumatic stress disorder OR trauma) in topic. |
| Google Scholar | 1.7.2020 | Google Scholar is used as a supplementary tool in this meta-analysis. Thus, we used the mostly commonly used combination (PTSD AND HIV) to search related to articles. |
| ProQuest | 12.10.2019 | We used ProQuest to search dissertations of master and doctor degrees. We randomly combined two key words from two sets of searching terms. The two sets of searching terms included participant- term (HIV OR AIDS) AND PTSD-terms (PTSD OR post-traumatic stress disorder OR trauma). |
| Reference lists | NA | We screened the reference lists from all included articles. In addition, we searched reference lists from previously published systematic review (Springer SA, Dushaj A, Azar MM. The impact of DSM-IV mental disorders on adherence to combination antiretroviral therapy among adult persons living with HIV/AIDS: a systematic review. AIDS Behav. 2012;16: 2119-43.). |

**Table 2 Study quality of included studies**

| Study name | 1) Define the source of information (survey, record review) | 2) List inclusion and exclusion criteria for exposed and unexposed subjects (cases and controls) or refer to previous publications | 3) Indicate time period used for identifying patients | 4) Indicate whether or not subjects were consecutive if not population-based | 5) Indicate if evaluators of subjective components of study were masked to other aspects of the status of the participants | 6) Describe any assessments undertaken for quality assurance purposes (e.g., test/retest of primary outcome measurements) | 7) Explain any patient exclusions from analysis | 8) Describe how confounding was assessed and/or controlled. | 9) If applicable, explain how missing data were handled in the analysis | 10) Summarize patient response rates and completeness of data collection | 11) Clarify what follow-up, if any, was expected and the percentage of patients for which incomplete data or follow-up was obtained |
| --- | --- | --- | --- | --- | --- | --- | --- | --- | --- | --- | --- |
| Boarts et al. 2006 | Yes | Yes | Yes | Unclear | No | Yes | Yes | Unclear | Unclear | Yes | Yes |
| Brown et al. 2019 | Yes | Yes | Yes | Yes | No | Yes | Yes | Yes | Yes | Yes | Yes |
| Delahanty et al. 2004 | Yes | Yes | Yes | Unclear | Unclear | Yes | Yes | Unclear | Unclear | Yes | No |
| Ebrahimzadeh et al. 2017 | Yes | Yes | Yes | Yes | No | Yes | Yes | Unclear | Unclear | Yes | Unclear |
| Halkitis 2014 | Yes | Yes | Yes | Yes | No | Yes | Yes | Yes | Yes | Yes | No |
| Keuroghlian et al. 2011 | Yes | Yes | Yes | Unclear | No | Yes | Yes | Unclear | Unclear | Yes | No |
| Negiet al. 2018 | Yes | Yes | Yes | Unclear | Yes | Yes | Yes | Yes | No | No | No |
| Schoennessonet al. 2017 | Yes | Yes | Yes | No | Yes | Yes | Yes | Yes | Unclear | Unclear | No |
| Sauceda 2014 | Yes | Yes | Yes | No | Unclear | Yes | Yes | Yes | Unclear | Unclear | No |
| Vranceanu et al. 2008 | Yes | Yes | Yes | Yes | Unclear | Yes | Yes | Unclear | Unclear | No | No |
| Wagner et al. 2012 | Yes | Yes | Yes | Yes | Unclear | Yes | Yes | Yes | Unclear | No | No |
| Whetten et al. 2013 | Yes | Yes | Yes | Yes | Unclear | Yes | Yes | Unclear | Yes | Yes | No |
